# Risk Factors Associated with Malnutrition in One-Year-Old Children Living in the Peruvian Amazon

**DOI:** 10.1371/journal.pntd.0003369

**Published:** 2014-12-11

**Authors:** Serene A. Joseph, Martín Casapía, Brittany Blouin, Mathieu Maheu-Giroux, Elham Rahme, Theresa W. Gyorkos

**Affiliations:** 1 McGill University, Department of Epidemiology and Biostatistics, Montréal, Québec, Canada; 2 Research Institute of the McGill University Health Centre, Division of Clinical Epidemiology, Montréal, Québec, Canada; 3 Asociación Civil Selva Amazónica, Iquitos, Peru; 4 Department of Global Health & Population, Harvard School of Public Health, Boston, Massachusetts, United States of America; 5 McGill University, Department of Medicine, Montréal, Québec, Canada; Case Western Reserve University School of Medicine, United States of America

## Abstract

**Background:**

Children under two years of age are in the most critical window for growth and development. As mobility increases, this time period also coincides with first exposure to soil-transmitted helminth (STH) infections in tropical and sub-tropical environments. The association between malnutrition and STH infection, however, has been understudied in this vulnerable age group.

**Methodology/Principal Findings:**

A nested cross-sectional survey was conducted in 12 and 13-month old children participating in a deworming trial in Iquitos, an STH-endemic area of the Peruvian Amazon. An extensive socio-demo-epi questionnaire was administered to the child's parent. Length and weight were measured, and the Bayley Scales of Infant and Toddler Development were administered to measure cognition, language, and fine motor development. Stool specimens were collected to determine the presence of STH. The association between malnutrition (i.e. stunting and underweight) and STH infection, and other child, maternal, and household characteristics, was analyzed using multivariable Poisson regression. A total of 1760 children were recruited between September 2011 and June 2012. Baseline data showed a prevalence of stunting and underweight of 24.2% and 8.6%, respectively. In a subgroup of 880 randomly-allocated children whose specimens were analyzed by the Kato-Katz method, the prevalence of any STH infection was 14.5%. Risk factors for stunting in these 880 children included infection with at least one STH species (aRR = 1.37; 95% CI 1.01, 1.86) and a lower development score (aRR = 0.97; 95% CI: 0.95, 0.99). A lower development score was also a significant risk factor for underweight (aRR = 0.92; 95% CI: 0.89, 0.95).

**Conclusions:**

The high prevalence of malnutrition, particularly stunting, and its association with STH infection and lower developmental attainment in early preschool-age children is of concern. Emphasis should be placed on determining the most cost-effective, integrated interventions to reduce disease and malnutrition burdens in this vulnerable age group.

## Author Summary

Malnutrition, including stunting and underweight, is one of the leading causes of morbidity and mortality in preschool-age children. Children under two years of age are at a particularly critical period for growth and development, and for first exposure to worm infections in tropical and subtropical environments. The association between malnutrition and worm infection, however, is not well understood in this age group. A nested cross-sectional survey was therefore conducted between September 2011 and June 2012 in 1760 children 12 and 13 months of age living in a worm-endemic area of the Peruvian Amazon. Length, weight, development (i.e. cognitive, language and motor development), worm infection, and socio-demographic information were obtained. Results showed a high prevalence of stunting, and a significant association with worm infection and lower development. Overall, these adverse effects have the potential to negatively impact short-term and long-term health and nutrition, and educational and social achievement, into school-age and adulthood. Emphasis is needed on determining the most appropriate and effective interventions to reduce poor health and nutrition outcomes in this age group.

## Introduction

Malnutrition is the leading cause of mortality in preschool-age children (i.e. children under five years of age) in low- and middle-income countries (LMICs). Over 150 million children suffer from one or more forms of malnutrition, including stunting, underweight and wasting [1], [2]. Malnutrition also predisposes to infection, creating a vicious infection-malnutrition cycle that contributes to over 35% of the disease burden of early childhood [1], [3]. Infection and micronutrient and other deficiencies from an inadequate diet are the primary causes of malnutrition in childhood [4]. Early childhood before the age of two years is a particularly critical time for growth faltering [5]. This window of time corresponds to weaning and the introduction of complementary foods. As mobility increases, the risk of early acquisition of certain infectious pathogens also increases during this time. The soil-transmitted helminths (STHs), or worm infections, are one such pathogen cluster that is transmitted through contaminated food, water and/or the environment in warm, tropical and subtropical climates. The STH disease cluster includes *ascariasis* (caused by the roundworm *Ascaris lumbricoides*), *trichuriasis* (caused by the whipworm *Trichuris trichiura*) and *ancylostomiasis* or hookworm disease (caused either by *Ancylostoma duodenale* or *Necator americanus*). The geographical distribution of these three diseases is overlapping, mainly in areas of poverty with poor sanitation and limited access to potable water. STHs are one of the most important Neglected Tropical Diseases (NTDs) and one of the most common infections worldwide. Recent estimates indicate that 1.45 billion people are infected with STHs in over 100 endemic countries [6]. It is estimated that they contribute 4.98 million years lived with disability (YLD) and 5.18 million disability-adjusted life years (DALYs) [6]. STHs are a significant contributor to poor health and nutritional status in all age groups, and especially in childhood.

Traditionally, the occurrence of STH infection had been perceived to be low in children under two years of age. However, there has been increasing empirical evidence which shows that the opposite is true [7]. In Belén, a community of extreme poverty in the Peruvian Amazon, while the prevalence of *Ascaris* or *Trichuris* was only 4% in children at seven to nine months of age, it rose to almost 30% at 12 to 14 months of age [8]. In a cohort of preschool-age children in Ecuador, over 20% suffered from *Ascaris* or *Trichuris* infection at least once in the first two years of life, with infection first appearing around seven months of age [9]. There is also evidence to suggest that hookworm infection may be high in early preschool-age children. A study in Zanzibar by Stoltzfus et al (2004), demonstrated that 31.3% of children under 30 months of age were infected with hookworm [10].

It is becoming increasingly recognized that STH infection in early childhood may have important adverse effects on health and nutrition [11], [12]. One such reason for this is that the parasites take up a greater proportion of the body in younger children [13]. However, the importance of STH infection and its link with malnutrition in preschool-age children has been inadequately studied. Few studies have included preschool-age children in their study population. Even fewer studies have provided age-disaggregated data to examine differing effects and sequelae in the critical growth window before two years of age. Evidence from the World Health Organization (WHO) Child Growth Standards demonstrates that, with appropriate nutrition and health interventions provided early in life, all children have a similar potential for healthy growth and development [14]–[16]; however, children living in areas of greatest poverty suffer the most from health and social inequities due to increased disease burden and lack of access to necessary health interventions and services [17]. Improving the health of the youngest children has been a focus of many international efforts, including Canada's Muskoka Initiative, and the Millennium Development Goals (MDGs) which aim to reduce poverty worldwide by 2015. With focus now shifting to the post-2015 MDG agenda, it is imperative to fill in knowledge gaps on the burden of disease and risk factors in early childhood to improve health in the short and the long term [18].

The principal objective of this study was to determine the association between malnutrition (i.e. stunting and underweight) and soil-transmitted helminth infection and other child, maternal and household characteristics in 12 and 13-month old children, living in an area of extreme poverty in the Peruvian Amazon.

## Methods

### Ethics approval

This study received ethics approval in Peru from the Comité Institucional de Ética of the Universidad Peruana Cayetano Heredia and the Instituto Nacional de Salud, in Lima, and the local Ministry of Health office (Dirección Regional de Salud Loreto) in Iquitos. Ethics approval was obtained in Canada from the Research Ethics Board of the Research Institute of the McGill University Health Centre in Montréal, Québec. Written informed consent was obtained by the parents or guardian of each child that participated in the study.

### Study population

**Figure 1:**
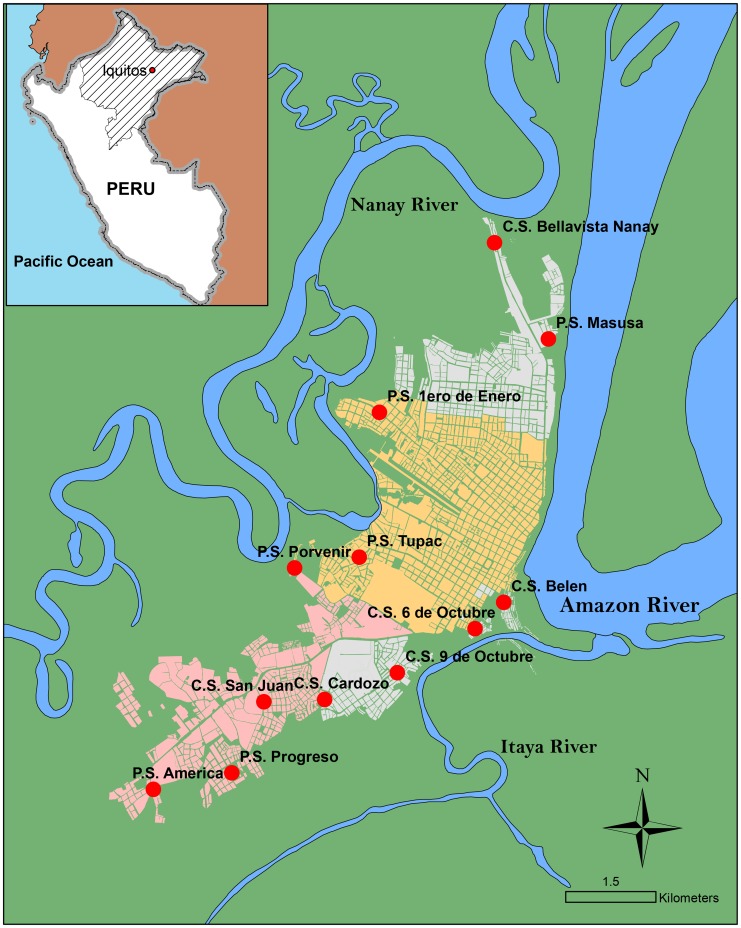
Map of the study area and location of the 12 participating health centres, Iquitos, Loreto, Peru. Enlarged area shows the city of Iquitos.

This study was conducted in neighbouring districts in and around the city of Iquitos, the capital of the Loreto region in the Peruvian Amazon (Fig. 1). The study area included four districts (Belén, Iquitos, Punchana and San Juan) where poverty is widespread, STH infections are highly endemic and malnutrition prevalence is high. Both malnutrition and STH prevalence have been identified as priority concerns by stakeholders in the community [19].

The study population included children attending their routine 12-month growth and development (“Crecimiento y Desarrollo” or CRED) clinic visit in the study area, and whose parents had agreed to their participation in a randomized controlled trial (RCT) to determine the benefit of deworming (mebendazole) on growth and development (ClinicalTrials.gov #NCT01314937). The current cross-sectional survey was nested within the deworming RCT, and describes information obtained at the baseline 12-month CRED trial visit.

Preschool-age children are scheduled to attend routine government-sponsored CRED visits (similar to well baby clinics) at health clinics in Peru once-monthly from birth to 11 months of age (with two visits before one month of age), and every two months from 12 to 24 months of age (with less frequent visits thereafter to school age). During routine CRED visits, anthropometric measurements (e.g. length and weight) are taken, developmental milestones are recorded, and children receive routine age-appropriate vaccinations and micronutrient supplements. Parents also receive nutrition and other health counselling for their child [20].

Using information provided by the Peruvian Ministry of Health on health centre location and attendance, 12 study health centres (“Centros de Salud” (C.S.) and “Puestos de Salud” (P.S.)) were identified in the study area. These included: 1) P.S. America; 2) C.S. Belen; 3) C.S. Bellavista Nanay; 4) C.S. Cardozo; 5) P.S. 1 de Enero; 6) C.S. 6 de Octubre; 7) C.S. 9 de Octubre; 8) P.S. Masusa; 9) P.S. Porvenir; 10) C.S. Progreso; 11) C.S. San Juan; and 12) P.S. Tupac Amaru.

Inclusion criteria for participating in the study were: 1) children attending any one of the study health centres for their 12-month CRED visit; and 2) children living in Belén, Iquitos, Punchana or San Juan districts. Exclusion criteria preventing participation in the study were: 1) children attending the health centre for suspected STH infection; 2) children who had received deworming treatment in the six months prior to the study; 3) children whose families planned to move outside of the study area within the next 12 months; 4) children under 12 months of age or 14 months of age or older; and 5) children with any serious congenital or chronic medical condition (e.g. chronic severe malnutrition, extremely preterm birth (i.e. < 28 weeks gestation), newborn hypoxia and neural tube defects). All inclusion and exclusion criteria were based on considerations related to participation in the deworming trial.

### Sample size

All children who were enrolled in the deworming RCT were included in the current study. The sample size of the RCT was estimated to be 1760 children, or 440 children per intervention group (MC4G Software©, GP Brooks, Ohio University, 2008).This was based on detecting a minimum difference of 0.20 kg in mean weight gain among different deworming interventions (3 intervention groups, and 1 control group).

### Recruitment

Canvassing of the local population was undertaken between April 2011 and August 2011 prior to recruitment to assist in identifying potentially eligible children for the study. In households where any child under 12 months of age was present, information was recorded on the child's date of birth and address. Lists of CRED attendance from each health centre were also provided to identify children who would be potentially eligible to participate in the study based on place of residence and age of the child.

Nine trained research assistants (RAs), primarily nurses and nurse-midwives, were assigned to one or two health centres each to recruit study participants in the respective communities and health centres and to obtain all study outcomes. Additional nurse-technicians were hired and trained to assist in participant recruitment. For parents of eligible children, an informed consent form was administered and signed. A questionnaire, which included questions on socio-demographic and health information about the child and family, was then administered by the RA during a household interview with that parent who was the primary caregiver. The questionnaire was adapted from previous studies [8], [21]–[23], but included additional questions related to child nutrition. These included history and duration of breastfeeding, and first introduction of liquids and solid foods. The latter was confirmed by redundancy among the questions and a 24-hour dietary recall. At the end of the home visit, parents were also provided with the information and materials needed to collect a stool specimen from the child. Parents were then given an appointment at the health centre, at which time they would deposit the stool specimen and the child's anthropometric measures and development would be ascertained. All forms and questionnaires were returned to the study offices at the end of each work day, and reviewed by the Project Director, the local Study Coordinator, and, when needed, by the local Principal Investigator, to confirm the eligibility of each child.

During the visit at the health centre, the quality of the stool specimen was first verified. If no specimen or an inadequate specimen (i.e. liquid specimen and/or insufficient quantity) was provided, then anthropometry was ascertained and a subsequent visit was scheduled to arrange for another stool specimen. If any child was discovered to be ill on the day of his or her health centre visit, the visit was postponed until the child had recovered. After verification of the quality and quantity of the stool specimen, the child was undressed and weighed (in duplicate) using a portable electronic scale (Seca 334, Seca Corp., Baltimore, MD, USA). Length (i.e. the recommended measurement for height in children less than two years of age) was measured (in duplicate) as recumbent crown-heel length on a flat surface using a stadiometer (Seca 210, Seca Corp., Baltimore, MD, USA). Cognition, receptive and expressive communication (i.e. language) and fine motor development were assessed using the Bayley Scales of Infant and Toddler Development, Third Edition (Bayley-III) (Pearson Education Inc, Texas, 2006). The latter instrument was translated into Spanish and adapted for local cultural appropriateness and validity by the Project Director (SAJ) and an experienced psychologist from the Instituto de Investigación Nutricional (IIN) in Lima, Peru. All RAs were trained on administration of the Bayley-III by SAJ and the IIN psychologist. As little variability was anticipated in gross motor skills at 12 months of age, and to reduce the length of time of assessment, the WHO gross motor milestones (i.e. walking alone, standing alone, walking with assistance, hands and knees crawling, and standing with assistance) was used instead of the Bayley-III Gross Motor subtest. Children's gross motor skill achievement was assessed by observation by RAs [24]. Upon completion of all baseline outcome measurements and the provision of an adequate stool specimen, participants were enrolled into the deworming trial and randomly assigned to one of three intervention groups or the control group. The stool specimen was labeled with a unique number between 1 and 1760, corresponding to the randomly assigned treatment code for the deworming trial.

Stool specimens were transferred to the laboratory at the local research facility (Asociación Civil Selva Amazónica) to be read by one of two experienced laboratory technologists. Two different techniques were required for reading stool specimens in the nested study based on the child's treatment allocation in the larger deworming RCT. Stool specimens from participants who were randomized to receive active deworming treatment were analyzed immediately by the Kato-Katz method, as recommended by WHO (within 24 hours of initial collection, as a fresh specimen is required for this technique) to determine both prevalence and intensity of STH infection [25], [26]. This procedure of immediately analyzing stool specimens only of those randomly allocated to the intervention groups receiving active deworming treatment takes into account the ethical imperative of treating those who would be found to have positive results. Stool specimens of those receiving inactive placebo tablets were stored in 10% formalin and examined by the direct method upon completion of the trial, at which time all participants received deworming treatment. To maintain blinding, each specimen code was replaced with a laboratory code by the local study coordinator for use by the laboratory technologists. Laboratory technologists were provided with a list of those laboratory codes which would be analyzed and those which were to be stored. Each list was kept on a password-protected computer, one in the coordinator's office and one in the lab accessible only to the laboratory supervisor. A master list linking all information was stored at the research office in Canada (Research Institute of the McGill University Health Centre). Quality control was conducted on 10% of all Kato-Katz slides to ensure agreement in species identification and egg counts between laboratory technologists.

### Statistical analyses

To classify child anthropometric measurements (i.e. length and weight) into categories of stunting, underweight and wasting, WHO Anthro software (Version 3, 2011) was used to calculate length-for-age z scores (LAZ), weight-for-age z scores (WAZ), and weight-for-length z scores (WLZ), respectively. Z scores are calculated taking into account a child's sex and age and are based on a comparison to a WHO international standard population. Moderate-to-severe categories of stunting, underweight and wasting are based on LAZ, WAZ and WLZ of <−2SD. Severe stunting, underweight and wasting are defined as LAZ, WAZ and WLZ of <−3SD, respectively [27].

Categories of STH infection intensity were determined from established WHO guidelines [28]. For *Ascaris* infection, light, moderate and heavy intensity are based on egg counts per gram of feces (epg) of 1–4999, 5000–49999 and 50000 and greater, respectively. For *Trichuris* infection, the categories for light, moderate and heavy intensity infection are an epg of 1–999, 1000–9999 and 10000 and greater, respectively. Light, moderate and heavy intensity hookworm infection are based on epgs of 1–1999, 2000–3999 and 4000 and greater, respectively. Both arithmetic and geometric mean epg were calculated and reported.

The development score was calculated as the mean crude score for each subtest of the Bayley-III, as well as a composite score of all four subtests combined. The range of possible scores was 0 to 91 for cognition, 0 to 49 for receptive communication, 0 to 48 for expressive communication and 0 to 66 for fine motor skills. Scaled scores were derived from scaling the total raw score in each individual subtest to a metric between 1 (i.e. the lowest possible score) and 19 (i.e. the highest possible score) according to the subtest and age of the child in months and days [29], [30]. As scaled scores are based on a study population that may not be representative of the general population, these scores were used for descriptive purposes only and not to quantify the level of developmental deficit. The WHO gross motor milestones were categorized into a dichotomous variable indicating whether the child had achieved the most advanced milestone of walking alone. The variable was coded as one, if the child was able to walk without any assistance or support, regardless of the other milestones achieved, and zero, if the child could not walk without assistance, but achieved at least one of the other gross motor milestones.

Principal Component Analysis was used to create an asset-based index for socioeconomic status (SES) to be included in multivariable analyses (StataCorp. 2013. *Stata Statistical Software: Release 13*. College Station, TX: StataCorp LP). Variables included in the index were house material, type of cooking fuel, television ownership, radio ownership and electricity in the home [31], [32]. The socioeconomic status index explained 40.1% of the variance and was divided into quartiles for subsequent analyses.

All associations with the outcomes of stunting and underweight were examined initially in univariable analyses. Variables with a p value<0.20, or that were deemed to be important from previous published research, were included in multivariable modelling to determine the most parsimonious model. If variables were highly correlated, the most informative variable (i.e. with more variation, more accurate measurements and/or important factors in previous literature) was chosen to be included in multivariable model building. Multivariable associations with stunting and underweight were examined using a generalized linear model with a log link, a Poisson distribution, and a robust variance estimator to estimate the risk ratio for the dichotomous outcomes of moderate-to-severe stunting and moderate-to-severe underweight, where no and mild categories of stunting, and no and mild categories of underweight, respectively, comprised the reference groups [33],[34]. Analyses were first restricted to children whose stool specimens were examined by the Kato-Katz method [26]. Analyses were then performed including all children in the study population. A complete case approach was used to analyze the data. All statistical analyses were performed using the Statistical Analysis Systems statistical software package version 9.3 (SAS Institute, Cary, NC, USA).

## Results

### Baseline characteristics of the study population

Between September 2011 and June 2012, parents of 2297 children 12 to 13 months of age were approached to participate in the study in order to meet the sample size requirements of 1760 eligible children. Three-hundred and eighty-five children did not meet the inclusion criteria, parents of 126 children declined to participate, and 26 children were recruited but the sample size was reached before they were enrolled in the study. Anthropometric and development measurements and stool specimens were obtained from all 1760 enrolled children. Baseline characteristics of the study population are described in Table 1. The average number of CRED visits before enrolment in the study (i.e. from birth to 11 months, inclusive) was 7.6 (±3.5). Less than 4% of children had no previous CRED attendance (n = 62). Only 25.5% (n = 447) had all vaccinations up-to-date according to Peruvian Ministry of Health guidelines (i.e. one dose of Bacille Calmette-Guérin (BCG), one dose of hepatitis B, three doses of polio, three doses of pentavalent, two doses of rotavirus, three doses of pneumococcal and one dose of measles, mumps and rubella (MMR) vaccines) [20]; however, as MMR vaccine and the third dose of pneumococcal vaccine are scheduled at the 12-month CRED visit, many children had not yet received these latter vaccinations. Including only vaccinations scheduled prior to 12 months, coverage of up-to-date vaccinations reached 80.3%. In terms of family and household characteristics, the average maternal age was 26.5 (±7.1) years. The average number of people living in the household was 6.6 (±2.7). Sixty-nine percent of children had one or more siblings. Roughly half of the children (50.1%) had received liquids (other than water and water-based drinks) or food before the age of six months. Baseline socio-demographic and epidemiological characteristics were similar in the 880 children whose stool specimens were examined by the Kato-Katz method compared to the entire study population of children (n = 1760) (results not shown).

**Table 1:** Prevalence of stunting and underweight in 12 and 13-month old children, by child, maternal and household characteristics, Iquitos, Peru, September 2011 to June 2012 (n = 1760).

	n	%	Prevalence of stunting (%)	Prevalence of underweight (%)
***Child characteristics***				
Age				
12 months	1586	90.1	23.1	8.6
13 months	174	9.9	33.9	8.6
Sex				
Male	920	52.3	28.8	10.2
Female	840	47.7	19.2	6.3
Birth weight*				
Low (<2500 g)	122	7.7	38.5	15.6
Normal (≥2500 g)	1472	92.4	22.5	7.5
Continued breastfeeding at 1 year				
Yes	1575	89.5	23.6	8.3
No	185	10.5	29.7	10.5
Up-to-date vaccinations*				
Yes**	1410	80.3	23.3	7.7
No	347	19.8	27.7	12.4
Received vitamin A in previous year				
Yes	921	52.3	21.9	6.7
No	839	47.7	26.7	10.7
Any hospitalizations since birth				
Yes	163	9.3	35.6	8.6
No	1597	90.7	23.0	8.6
Cognitive development scaled score†				
0–9	677	38.5	31.8	14.0
10–19	1083	61.5	19.5	5.3
Receptive communication scaled score†				
0–9	1533	87.1	25.1	9.7
10–19	227	12.9	18.5	1.8
Expressive communication scaled score†				
0–9	1494	84.9	25.9	9.5
10–19	266	15.1	14.7	3.8
Fine motor skills scaled score†				
0–9	1050	59.7	29.0	10.7
10–19	710	40.3	17.2	5.6
Walking alone				
Yes	433	24.6	14.3	3.5
No	1324	75.4	27.4	10.3
***Maternal characteristics***				
Marital status				
Married/common-law	1423	80.9	24.2	8.9
Single	337	19.2	24.0	7.7
Highest level of education*				
Secondary incomplete	1205	68.5	27.6	10.1
Secondary complete	554	31.5	16.8	5.4
Employment outside of home				
Yes	179	10.2	31.3	6.2
No	1581	89.8	23.4	8.9
Place of delivery				
Health establishment	1591	90.4	24.1	8.1
Home	169	9.6	24.9	13.6
Attended any antenatal care				
Yes	1658	94.2	24.0	8.1
No	102	5.8	27.5	17.7
***Household characteristics***				
Socioeconomic status (SES)				
First quartile (i.e. lowest)	423	24.0	28.1	11.6
Second quartile	458	26.0	25.1	10.9
Third quartile	419	23.8	27.5	7.9
Fourth quartile (i.e. highest)	460	26.1	16.7	4.4
District				
Urban	200	11.4	21.5	6.0
Peri-urban/Rural	1560	88.6	24.6	9.0

*Totals do not sum to 1760 due to missing responses on birth weight (n = 166 missing), vaccinations (n = 3 missing) and maternal education (n = 1 missing).

**Up-to-date vaccinations include those scheduled between birth and 11 months of age (i.e. one dose of Bacille Calmette-Guérin (BCG), one dose of hepatitis B, three doses of polio, three doses of pentavalent, two doses of rotavirus, and two doses of pneumococcal).

† Scaled development scores are derived from the total raw score in each individual subtest, scaled between 1 and 19 (with a mean of 10) according to the subtest and the age of the child in months and days [29].

### Study population profile of malnutrition, STH infection and development

Twenty-five percent of the study population suffered from one or more forms of malnutrition. Prevalence of moderate-to-severe underweight, stunting and wasting were 8.6%, 24.2% and 2.3%, respectively (Table 2). Co-morbidity with two or three concurrent forms of malnutrition was present in 8.3% (n = 146) of participants. Mean z scores for the study population were below average (i.e. below 0) for all three indices (i.e. LAZ, WAZ and WHZ). Severe malnutrition (i.e. a z score of <−3 SD for LAZ, WAZ or WLZ) affected 5.5% (n = 96) of the population.

**Table 2:** Nutritional indicators in 12 and 13-month old children, Iquitos, Peru, September 2011 to June 2012 (n = 1760).

Nutritional Indicator		
Weight (kg) and derived indices*		
Weight [mean (SD**)]	8.72	(0.98)
Weight-for-age Z score [mean (SD)]	−0.73	(0.96)
Moderate-to-severe underweight [% (n)]	8.6	(152)
Severe underweight [% (n)]	1.4	(25)
Length (cm) and derived indices**		
Length [mean (SD)]	72.13	(2.44)
Length-for-age Z score [mean (SD)]	−1.36	(0.96)
Moderate-to-severe stunting [% (n)]	24.2	(426)
Severe stunting [% (n)]	4.9	(86)
Weight (kg)/length (cm) and derived indices**		
Mean weight-for-length Z score	−0.10	(0.92)
Moderate-to-severe wasting [% (n)]	2.3	(40)
Severe wasting [% (n)]	0.2	(3)

*Using WHO international growth standards [14].

**SD = standard deviation.

The overall prevalence of any STH infection in children whose stool specimens were analyzed by the Kato-Katz method was 14.5% (Table 3). The prevalence of infection was 11.5% for *Ascaris*, 4.5% for *Trichuris* and 0.6% for hookworm. Eighteen children (2.1%) were infected with two STH species, but none with all three. For those who had their stool specimens stored and analyzed by the direct method, the prevalence was lower for all three STH species (i.e. 9.5%, 0.9% and 0.1% for *Ascaris, Trichuris*, and hookworm, respectively). Using the Kato-Katz method as the gold standard, and assuming equal STH prevalence due to randomization, the direct method, therefore, underestimated *Ascaris* infection by 17.4%, *Trichuris* infection by 80.0%, hookworm infection by 83.3%, and any STH prevalence by 29.0%. For the 880 children whose stool specimens were examined using the Kato-Katz method and who were found to be STH positive, most were found to have low intensity infection, with 86.1%, 92.5% and 100% harbouring light infections of *Ascaris*, *Trichuris* and hookworm, respectively (Table 4). There were no cases of heavy intensity infection of any STH species.

**Table 3:** Soil-transmitted helminth (STH) prevalence in 12 and 13-month old children, Iquitos, Peru, September 2011 to June 2012, by stool examination method.

		Prevalence			
		Kato-Katz*1 (n = 880)		Direct method*2 (n = 880)	
		% (n)		% (n)	
*Ascaris*	Infected	11.5	(101)	9.5	(84)
	Not infected	88.5	(779)	90.5	(796)
*Trichuris*	Infected	4.5	(40)	0.9	(8)
	Not infected	95.5	(840)	99.1	(872)
Hookworm	Infected	0.6	(5)	0.1	(1)
	Not infected	99.4	(875)	99.9	(879)
Any STH	Infected	14.5	(128)	10.3	(91)
	Not infected	85.5	(752)	89.7	(789)

*^1^The Kato-Katz method was used to analyze fresh stool specimens of those receiving deworming at baseline.

*^2^The direct method was used to analyze stored stool specimens of those receiving placebo at baseline (i.e. average of 17 months later).

**Table 4:** Soil-transmitted helminth (STH) intensity in 12 and 13-month old children, Iquitos, Peru, September 2011 to June 2012, using the Kato-Katz method (n = 880).

	Intensity* (% (n))						Mean epg**			
	Light		Moderate		Heavy		AM***1 (95% CI)		GM***2 (95% CI)	
*Ascaris*	86.1	(87)	13.9	(14)	0	(0)	288.3	(195.9, 380.8)	2.2	(1.9, 2.6)
*Trichuris*	92.5	(37)	7.5	(3)	0	(0)	18.1	(5.5, 30.6)	1.3	(1.2, 1.3)
Hookworm	100.0	(5)	0	(0)	0	(0)	2.0	(0.8, 3.3)	1.0	(1.0, 1.1)

*Intensity data available only for those receiving deworming at baseline (i.e. n = 880 specimens analyzed by the Kato-Katz method).

**epg = eggs per gram. The calculation of mean epg includes infected and uninfected individuals.

***^1^AM = arithmetic mean;

***^2^GM = geometric mean.

A value of 1 was added to each observation to calculate the geometric mean.

In terms of developmental functioning in all 1760 children, the mean composite development score on the Bayley-III was 98.1 (± SD 6.0) with a range between 73 and 123 points. On individual subtests, the mean score was 42.5 (±3.0) for cognition, 12.9 (±1.6) for receptive communication, 13.5 (±2.1) for expressive communication and 29.2 (±1.5) for fine motor skills. This translated to a mean scaled score of 9.9 (±1.84), 7.2 (±1.9), 8.1 (±1.7) and 9.2 (±1.5) for the cognitive, receptive language, expressive language and fine motor subtests, respectively. The mean scores were slightly higher for 13-month old children compared to 12-month old children (i.e. 43.2 vs. 42.5 for cognition, 13.3 vs. 12.9 for receptive communication, 13.8 vs. 13.4 for expressive communication, and 29.4 vs. 29.2 for fine motor skills, respectively). Twenty-three percent and 35.6% of 12 and 13-month old children, respectively, were able to walk without support.

### Risk factors for stunting and underweight

In determining the risk factors for malnutrition in the group of children whose specimens were analyzed by the Kato-Katz method, stunting was found to be statistically significantly associated with the presence of any STH infection, male sex, older age (i.e. 13 months old), one or more hospitalizations since birth, lower SES, and lower birth weight in both unadjusted and adjusted analysis (Table 5). The crude score of each individual Bayley-III subtest was significantly associated with stunting in univariable analyses. The overall composite development score was included in the multivariable model, with a lower score associated with an increased risk of stunting in the adjusted model (aRR 0.97; 95% CI: 0.95, 0.99).

**Table 5:** Risk factors for stunting and underweight in 12 and 13-month old children in Iquitos, Peru, September 2011 to June 2012 (n = 796*).

Child, maternal and household characteristics	Stunting**		Underweight**	
	Crude RR*** (95% CI)	Adjusted RR‡1 (95% CI)	Crude RR (95% CI)	Adjusted RR‡2 (95% CI)
Any STH infection (yes vs. no)	1.32 (0.99, 1.76)	1.37 (1.01, 1.86)	1.24 (0.73, 2.10)	1.15 (0.65, 2.03)
Sex (male vs. female)	1.36 (1.07, 1.71)	1.35 (1.07, 1.72)	1.52 (1.00, 2.31)	NS‡
Age (13 vs. 12 months)	1.45 (1.06, 2.00)	1.52 (1.09, 2.12)	0.47 (0.18, 1.26)	NS
Birth weight (per kg increase)	0.44 (0.36, 0.55)	0.48 (0.38, 0.59)	0.38 (0.26, 0.54)	0.43 (0.30, 0.63)
Continued breastfeeding at 12 months (no vs. yes)	1.32 (0.96, 1.81)	NS	1.51 (0.87, 2.61)	1.73 (1.00, 2.98)
Vaccinations up-to-date† (no vs. yes)	1.27 (0.98, 1.64)	NS	1.67 (1.08, 2.59)	NS
Vitamin A supplementation in previous year (no vs. yes)	1.27 (1.01, 1.59)	NS	1.87 (1.24, 2.84)	NS
Any hospitalization since birth (yes vs. no)	1.77 (1.32, 2.37)	1.54 (1.12, 2.11)	NS	NS
Mean development (per 1 point increase)§	0.96 (0.94, 0.97)	0.97 (0.95, 0.99)	0.91 (0.89, 0.94)	0.92 (0.89, 0.95)
Walking alone (yes vs. no)	0.63 (0.46, 0.85)	NS	0.46 (0.26, 0.84)	NS
First vs. fourth SES∥ quartile	2.01 (1.38, 2.92)	1.62 (1.11, 2.35)	3.89 (1.83, 8.28)	2.85 (1.28, 6.32)
Second vs. fourth SES quartile	1.75 (1.19, 2.56)	1.60 (1.10, 2.32)	3.30 (1.53, 7.10)	2.58 (1.18, 5.65)
Third vs. fourth SES quartile	2.01 (1.38, 2.92)	1.59 (1.10, 2.30)	2.55 (1.15, 5.66)	2.17 (0.99, 4.78)
Maternal employment outside of home (no vs. yes)	1.24 (0.90, 1.71)	NS	NS	NS
Place of residence (periurban/rural vs. urban)	NS	NS	2.31 (0.96, 5.58)	NS
Place of delivery (home vs. hospital)	NS	NS	1.87 (1.10, 3.16)	NS
Antenatal care attendance (no vs. yes)	NS	NS	2.07 (1.11, 3.87)	NS

*The analysis was restricted to the 880 children whose stool specimens were analyzed by the Kato-Katz method. The adjusted models include a sample size of 796 due to 84 missing responses on birth weight.

**Reference group includes mild or no stunting (i.e. LAZ≥−2 SD) and mild or no underweight (i.e. WAZ≥−2 SD).

***RR = risk ratio: Crude results include variables with a p level<0.20 for model-building purposes.

‡1 RR for stunting adjusted for any STH infection, sex, age, birth weight, any hospitalizations since birth, development score and socioeconomic status;

2 RR for underweight adjusted for any STH infection, birth weight, continued breastfeeding, development score and socioeconomic status.

‡ NS = not statistically significant.

† Up-to-date vaccinations include those scheduled between birth and 11 months of age (i.e. one dose of Bacille Calmette-Guérin (BCG), one dose of hepatitis B, three doses of polio, three doses of pentavalent, two doses of rotavirus, and two doses of pneumococcal vaccines).

§ Mean development score is the combined sum of the raw scores of each individual subtest of the Bayley-III.

∥ SES = socioeconomic status (lowest quartile = lowest SES; highest quartile = highest SES).

Risk factors for underweight in unadjusted and adjusted analyses included lower birth weight, lower development score, and lower SES (Table 5). Continued breastfeeding at one year of age was associated with a decreased risk of underweight in unadjusted and adjusted analyses. No statistically significant association was found between underweight and any STH infection in either unadjusted or adjusted analyses.

No independent associations were found between malnutrition and up-to-date vaccinations, vitamin A supplementation, walking alone, maternal employment outside of the home, place of residence, place of delivery or antenatal care attendance (Table 5). The timing of introduction of liquids and foods was not associated with stunting or underweight in either unadjusted or adjusted analyses. STH infection was not associated with wasting in either unadjusted or adjusted analyses (results not shown).

Multivariable results for stunting, underweight and wasting were similar when analyses were extended to include participants with specimens analyzed by both the Kato-Katz and the direct method (results not shown).

## Discussion

The scientific literature to date has provided insufficient evidence of an association between malnutrition and STH infection in early preschool-age children. This nested cross-sectional study in 1760 preschool-age children aged 12 and 13 months in a community of extreme poverty in the Peruvian Amazon contributes to filling this research gap. We demonstrate an important association between malnutrition and STH infection and developmental deficits. Previous studies in the area of Belen have found similar associations between malnutrition and STH infection in a wider age range of preschool-age children [8], [23]. In contrast to previous studies, however, this association was apparent even with low intensity STH infection [8]. The current study updates previous estimates and provides in-depth data for that critical time period around one year of age when interventions are likely to be considered to be integrated into vaccination programs or well baby clinics. Consistent with previous studies, lower socioeconomic status and older child age were associated with a higher risk of malnutrition [8], [23], [35]. Nonetheless, the latter result is somewhat unexpected, as the age range was quite restricted in the present study. This finding, along with a greater number of children who were walking alone at 13 months of age, support the concept of a critical window in which children are rapidly developing and growing before two years of age [5]. This has the potential to translate to an even greater impact of parasite infection and nutritional deficits on child health in this time period.

An interesting finding in this study was that STH prevalence (from either detection method) and malnutrition prevalence were lower compared to previous work in the area [8]. The current study was embedded within the existing health infrastructure of routine growth and development clinic visits. Although previous attendance was not an inclusion criterion, there may have been higher-risk populations with low CRED attendance that would not have been easily reached, but who may have been included in the previous community-based surveys. We attempted to solve this problem by conducting community canvassing prior to enrolment to identify all children in the eligible jurisdictions, not only those who had had the opportunity to access health services previously. An increase in research attention and community-based health and nutrition campaigns may also explain some of the improvements. In particular, deworming campaigns directed towards school-age children, may have contributed to a reduction in overall environmental contamination in the area. This could have resulted in lower infection rates in younger children not directly targeted by campaigns, as has been shown in other settings [36]. A recent study also demonstrated a decrease in the prevalence of stunting in preschool-age children in Peru from 1991 to 2011, possibly due to economic growth and an increased emphasis on pro-poor social programs [37]. However, the overall prevalence of stunting has remained unacceptably high, with children between 12 and 23 months, those living in the Amazon or Andean region, and those of lower SES, suffering disproportionately from malnutrition [37]. Prevalence of stunting was also higher in males compared to females under the age of 36 months, which is consistent with our findings. Despite the positive trends in a reduction in stunting and STH infection in this and other studies, the current results demonstrate that even low STH prevalence and intensity of infection can be associated with poor growth in children in this vulnerable age group.

This study benefits from a large sample size of children, representative of the wider population of children living in the STH high-risk flooding areas of Iquitos. This representativity was helped in part by the community-wide canvassing and by the inclusion of health centres from a wide catchment area. Nevertheless, hard-to-reach and hidden populations of children suffering from severe malnutrition or other chronic illnesses may be under-represented in the study. An additional strength of the study is the focus on children of a narrow age range in the critical growth window. Other studies have included populations of children at heterogeneous growth and development stages and have been unable to disaggregate outcome results by age.

In-depth information on potential risk factors was also collected to ensure that the impact of other child, maternal and household characteristics were taken into account in the analysis. The ascertainment of nutritional information, such as when liquids and foods were first introduced and the age of weaning may have been limited by recall bias; however, the collection of information on the age of introduction of specific local foods and a 24-hour recall were used to increase validity of the responses. This study also incorporated comprehensive developmental testing. To our knowledge, this is the first study that has incorporated the Bayley-III, one of the most rigorous development tests available for preschool-age children, in conjunction with STH infection. We were also able to take into account the potential effects of SES by using an asset-based proxy index.

The study was limited by the fact that, for ethical reasons, the Kato-Katz method could only be used to analyze half of the specimens from randomly-allocated participants (i.e. those who were randomly assigned to receive active deworming treatment), and therefore intensity data were not available for all participants. The higher STH prevalence in specimens analyzed by the Kato-Katz technique suggests that the direct method likely underestimated STH prevalence, due to lower sensitivity and specificity, and/or the storage of specimens. In those with intensity data, a low prevalence of moderate-to-heavy intensity infection restricted the ability to detect differences in malnutrition risk according to the intensity of STH infection. In addition, although the malnutrition-infection association is known to be cyclical in nature [1], [3], the direction of the associations between various risk factors and malnutrition cannot be established due to the cross-sectional nature of the baseline survey. One additional limitation concerns the classification of development scores and their potential for generalization to other populations. These should be interpreted within the context of the current study population and would require further validation for use and for comparisons with populations in other settings'.

Overall, this study demonstrates an important association between stunting, low birth weight, SES, STH infection and cognitive, language and motor development in early preschool-age children. This empirical evidence advances our knowledge of the risk factors for malnutrition in the critical growth and development window before two years of age. The results provide further evidence of the importance of determining the most cost-effective, integrated and multi-sectoral interventions to target this vulnerable age group, reduce health inequities, and prevent growth and development deficits in both the short and long-term.

## Supporting Information

Checklist S1 STROBE Checklist.
(DOC)
